# The Effect of Ethanol Extract from *Mesua ferrea* Linn Flower on Alzheimer’s Disease and Its Underlying Mechanism

**DOI:** 10.3390/cimb45050259

**Published:** 2023-05-06

**Authors:** Kusawadee Plekratoke, Chantana Boonyarat, Orawan Monthakantirat, Natsajee Nualkaew, Jinda Wangboonskul, Suresh Awale, Yaowared Chulikhit, Supawadee Daodee, Charinya Khamphukdee, Suchada Chaiwiwatrakul, Pornthip Waiwut

**Affiliations:** 1 Biomedical Science Program, Graduate School, Khon Kaen University, Khon Kaen 40002, Thailand; kusawadee2535@gmail.com; 2 Faculty of Pharmaceutical Sciences, Khon Kaen University, Khon Kaen 40002, Thailand; chaboo@kku.ac.th (C.B.); oramon@kku.ac.th (O.M.); nnatsa@kku.ac.th (N.N.); yaosum@kku.ac.th (Y.C.); csupawad@kku.ac.th (S.D.); charkh@kku.ac.th (C.K.); 3 Faculty of Pharmaceutical Sciences, Thummasart University, Bangkok 10330, Thailand; jindawangboonskul@gmail.com; 4 Division of Natural Drug Discovery, Institute of Natural Medicine, University of Toyama, 2630 Sugitani, Toyama 930-0194, Japan; suresh@inm.u-toyama.ac.jp; 5 Department of English, Faculty of Humanities and Social Sciences, Ubon Ratchathani Rajabhat University, Ubon Ratchathani 34000, Thailand; suchadachai65@gmail.com; 6 Faculty of Pharmaceutical Sciences, Ubon Ratchathani University, Ubon Ratchathani 34190, Thailand

**Keywords:** antioxidant, acetylcholinesterase inhibition, beta-amyloid aggregation, neuroprotection, apoptotic pathway, amyloidogenic pathway

## Abstract

The effects of *Mesua ferrea* Linn flower (MFE) extract on the pathogenic cascade of Alzheimer’s disease (AD) were determined by an in vitro and cell culture model in the search for a potential candidate for the treatment of AD. The 2,2′-azino-bis-3-ethylbenzthiazoline-6-sulphonic acid (ABTS) and 1,1-diphenyl-2-picrylhydrazyl (DPPH) assay exhibited that the MFE extract had antioxidant activities. According to the Ellman and the thioflavin T method’s result, the extracts could inhibit acetylcholinesterase and β-amyloid (Aβ) aggregation. Studies on neuroprotection in cell culture found that the MFE extract could reduce the death of human neuroblastoma cells (SH-SY5Y) caused by H_2_O_2_ and Aβ. Western blot analysis exhibited that the MFE extract alleviated H_2_O_2_-induced neuronal cell damage by downregulating the pro-apoptotic proteins, including cleaved caspase-3, Bax, and by enhancing the expression of anti-apoptotic markers including MCl_1_, BCl_xl_, and survivin. Moreover, MFE extract inhibited the expression of APP, presenilin 1, and BACE, and increased the expression of neprilysin. In addition, the MFE extract could enhance scopolamine-induced memory deficit in mice. Overall, results showed that the MFE extract had several modes of action related to the AD pathogenesis cascade, including antioxidants, anti-acetylcholinesterase, anti-Aβ aggregation, and neuroprotection against oxidative stress and Aβ. Therefore, the *M. ferrea* L. flower might be a possibility for further development as a medication for AD.

## 1. Introduction

Alzheimer’s disease (AD) is a neurodegenerative disorder causing dementia that is related to cognition and memory. The most common sites of pathology are the hippocampus and cerebral cortex [1]. According to World Alzheimer’s Report 2018, dementia cases including AD were estimated to be 50 million worldwide. Furthermore, they were likely to be doubled by 2030 and triple by 2050. In 2018, twenty people turned out to be diagnosed with dementia every minute. Dementia globally cost 1 trillion US dollars in 2018 and was anticipated to reach 2 trillion US dollars in 2030 [2]. The pathogenesis of AD is not yet fully understood, but the scientific consensus is certain that it is a complex disease brought on by various factors. These include aggregation of Aβ, deposition of neurofibrillary tangles (NFTs), deficiency of cholinergic neurotransmission, oxidative stress, and neuroinflammation. It has been shown that simultaneous cholinergic importance plays a role in the development of AD. Acetylcholine (ACh), which is crucial for cognitive function, is present in decreased concentrations and functions in AD patients. These anomalies, such as cholinergic neuron loss and decreased acetylcholinesterase activity, lend support to the cholinergic theory of AD [3]. In the aging brain, misfolded protein accumulates and leads to metabolic loss, oxidative stress-induced damage, and synapse dysfunction. Oxidative damage in AD is indicated by high levels of DNA oxidation products in mitochondria and nuclei [4,5]. Presently, the US Food and Drug Administration (FDA) has approved five drugs in three sub-classes for the treatment of AD including acetylcholinesterase (AChE) inhibitor (rivastigmine, galantamine, and donepezil), N-methyl D aspartate receptor (NMDA) antagonist (memantine), and a combined drug between AChE inhibitor and NMDA receptor antagonist (Namzaric^®^) [6]. However, the single target drug benefits in only the palliative treatment but has no effect of curing or preventing AD. Due to AD having several etiologies, the conventional strategy of modifying one target may not be enough to treat this complicated disease. Nowadays, a great source for potential therapies in various complex diseases is natural products such as herbs and plants. Therefore, it should be of great interest to find a new potential drug from natural products for AD treatment.

*Mesua ferrea* Linn, a species in Calophyllaceae family, is widely distributed in tropical countries such as India, Thailand, China, and New Guinea. Some important ayurvedic formulations contain *M. ferrea* L. such as Brahma rasayana, which can enhance cognitive function and improve memory [7]. Several studies showed that the *M. ferrea* L. exhibits several therapeutic actions including antioxidant [8], anti-microbial [9], anti-cancer [10], and anti-inflammation [11]. In addition, the crude extract of *M. ferrea* L. flower exhibits activities related to the pathogenesis of AD, which includes antioxidant [12] and anti-inflammation [11]. Several phytochemical studies have shown that the major chemical components of *M. ferrea* L. are phenolic compounds [13], flavonoids [13], coumarins [14], and xanthones [15]. From the literature review, these components are naturally occurring phytochemicals with heterocyclic structures that display a wide range of biological activities related to AD including anti-acetylcholinesterase, anti-Aβ aggregation, antioxidant, and anti-inflammation. Therefore, the aim of this study is to investigate the potential of MFE extract as an AD treatment. The MFE extract was investigated for the activities associated with AD including antioxidant, AChE function, Aβ aggregation, and neuroprotection. To clarify the mechanism of action, the expression levels of proteins involved with AD pathogenesis were determined by Western blotting analysis. Finally, the effects of the MFE extract on cognitive function were evaluated in a scopolamine-induced cognitive-deficit animal model.

## 2. Materials and Methods

### 2.1. Chemicals and Reagents

The flower powder of *M. ferrea* L. was offered by Chao Phya Abhaibhubejhr Hospital, Prachinburi Province, Thailand. The plant was identified by Benjawan Leenin, chief of the Traditional Knowledge Center, Chao Phya Abhaibhubejhr Hospital Foundation. The voucher specimen was deposited at the museum of Chao Phya Abhaibhubejhr Hospital. Quercetin, gallic acid, trolox, acetylthiocholine iodide (ATCI), tacrine, Aβ_1–42_, N-acetyl cysteine (NAC), Dulbecco’s modified Eagle medium nutrient mixture F-12 (DMEM/F12) trypsine, and fetal bovine serum (FBS), were purchased from Gibthai (GT Chemical supplies Co., Ltd., Bangkok, Thailand), Sigma-Aldrich (SM Chemical supplies Co., Ltd., Bangkok, Thailand), Fluka (SM Chemical supplies Co., Ltd., Bangkok, Thailand), and Merck (Merck, Bangkok, Thailand).

### 2.2. Plant Extraction

The flower powder of *M. ferrea* L. (3 kg) was extracted with ethanol for 7 days at room temperature. Then, the extract was dried in a rotary evaporator at reduced pressure at 45 °C after filtering to give the crude extract and kept in a refrigerator at 4 °C until needed for analysis.

### 2.3. Determination of the Total Phenolic and Flavonoid Contents

#### 2.3.1. Determination of the Total Phenolic Content

The total phenolic content was assessed using the Folin–Ciocalteu test. The method was performed as described by Blainski with minor modifications. Briefly, the MFE extract was dissolved with ethanol and added with 10% Folin–Ciocalteu reagent. Then, the mixture was added with 7.5% Na_2_CO_3_ and incubated for 2 h. After that, the absorbance of the mixture was detected at 700 nm. Total phenolic content was exhibited as gallic acid equivalents per milligram of extract (µg of GAE/mg) [16].

#### 2.3.2. Determination of the Total Flavonoid Content

Total flavonoid content using quercetin as standard. The assay was performed as described by Chang with minor modifications. The assay was carried out in 96-well plate by mixing the following: ethanol extract, 25 g/L of AlCl_3,_ and 100 g/L of CH_3_COONa. The mixture solution was incubated for 15 min. After incubation, the absorbance of the mixture solution was measured at 450 nm. The results were presented as quercetin equivalents per milligram of extracts (µg of QC/mg) [17].

### 2.4. In Vitro Assay for Activities Related to AD Pathogenesis

#### 2.4.1. Antioxidant Activity by DPPH and ABTS Assay

The DPPH assay was performed as directed by Songsiang. Briefly, 100 µL of the MFE extract was combined with 100 µL of 0.2 mM DPPH solution at room temperature for 30 min. After incubation, using a microplate reader, the absorbance was measured at 550 nm. The activity of the MFE extract to scavenge free radicals was evaluated by comparing its absorbance with that of a control. Antioxidant standard used was trolox [18].

The 2,2′-azinobis-(3-ethylbenzothiazoline-6-sulfonic acid) (ABTS^•+^) radical cation was decolonized to measure the antioxidant activity. The reaction of 7 mM ABTS with 2.45 mM potassium persulfate in water at ambient temperature and darkness for 12–16 h generated the ABTS^•+^. The ABTS^•+^ solution was diluted with ethanol prior to the experiment to obtain an absorbance of 0.70 ± 0.02 at 700 nm. In the experiment, the MFE extract (50 µL) was added to react with ABTS^•+^ solution (100 µL) for 15 min. After incubation, a measurement of the solution’s absorbance was performed at the wavelength of 700 nm. As a standard for antioxidants, trolox was used [19].

#### 2.4.2. Acetylcholinesterase Inhibitory Activity Assay

The MFE extract was evaluated for acetylcholinesterase (AChE) inhibitory activity by using the modified Ellman’s method. The experiment was prepared in 96-well plate by mixture with 25 μL of the MFE extract or standard (tacrine), 25 μL of 1 mM acetylthiocholine iodide (ATCI), 125 μL of 1 mM 5, 5′-dithiobis-(2 nitrobenzoic acid) (DTNB), and 50 μL of 0.2 U/mL AChE form an electric eel (type VI-S), respectively. The absorbance of enzyme activity was measured using the microplate reader at 405 nm and detected every 30 s for 5 min [20].

#### 2.4.3. Amyloid Aggregation Inhibitory Activity Assay

The MFE extract was investigated for anti-Aβ aggregation by using the thioflavin-T (ThT) assay. Briefly, 2 µL of the MFE extract or standard (curcumin) was incubated with 18 µL of 10 µM Aβ_1–42_ in 0.5 M phosphate buffer (pH 7.4) for 48 h at 37 °C. After incubation, 180 µL of 5 µM ThT in a glycine/NaOH solution (pH 8) was added to the sample and incubated for 5 min. Then, the fluorescence intensities in each well were recorded at wavelengths of 446 nm (excitation) and 490 nm (emission) using a fluorescence spectrophotometer [21].

### 2.5. Neuroprotective Activities Assay

Neuroblastoma cells (SH-SY5Y) were cultured in DMEM/F12 supplemented with 10% FBS in a humidified 5% CO_2_ incubator at 37 °C. SH-SY5Y cells were regularly cultured in a cell culture flask of 75 cm^2^ for 24 h. After that, the cells were added with 1% FBS in a culture medium and supplemented with 10 μM RA for 6 days to differentiate neural cells. The media replenished with RA were changed every 3 days [22]. Before testing, cells were plated in 96-well plates at a density of 5 × 10^5^ cells/mL and given an additional 48 h of incubation. The neuroprotective effect of MFE extract against H_2_O_2_-induced cell damage was determined using the cell-based assay. The differentiated SH-SY5Y cells were pretreated with the extract or standard (n-acetylcysteine, NAC) for 2 h. Then, cells were induced by 250 µM H_2_O_2_ for 2 h to generate oxidative stress conditions. The cell viability was determined by incubating the cells with 0.5 mg/mL MTT for 2 h and measured by a microplate reader at 550 nm [23].

For investigation of the neuroprotective effect against Aβ_1–42_-induced cell damage, preparing for the Aβ_1–42_ was modified from Takomthong et al. Aβ_1–42_ that had been lyophilized was reconstituted in PBS at a concentration of 250 μM and kept at −20 °C until analysis. Aliquots were diluted to a final concentration of 25 µM using a culture-free serum medium and incubated at 37 °C for 72 h to generate aggregated amyloid. For assays, the cells were incubated with MFE extract or standard (curcumin) for 2 h and after that, induced with aggregated 25 µM of Aβ_1–42_ for 24 h. The cell viability was evaluated by MTT assay [23].

### 2.6. Effect on the Expression of AD- and Apoptosis-Related Proteins in the SH-SY5Y Cells

The differentiated SH-SY5Y cells were plated into a 6-well plate at a concentration of 1 × 10^6^ cell/mL for 48 h. Then, the cells were treated with MFE extract or standard (NAC) for 2 h. After that, the cells were induced with 250 µM of H_2_O_2_ for 8 min. Regarding the procedure for lysing cells, lysis buffer was added to the treated cells for 30 min. The lysate was centrifuged for 10 min at 4 °C at 13,500 rpm, and the supernatants were collected. After that, the Bradford test was used to measure the total protein content. The SDSPAGE method was used to separate proteins and transferred them to a polyvinylidene difluoride (PVDF) membrane. The membrane was probed with primary antibodies including APP, presenilin 1, beta-site APP cleaving enzyme (BACE), neprilysin, survivin, bax, bcl-xl, mcl-1, cleave caspase 3, and actin. After that, secondary antibodies were allowed to incubate on the membranes. Finally, the technology for enhanced chemiluminescence was used to see the membranes [24].

### 2.7. The Effects of MFE Extract on Scopolamine-Induced Memory Impairments in Mice

The mice ICR 6 weeks old weighing between 30 and 35 g were derived from the Northeast Laboratory Animal Center, Khon Kaen University. All animal experiments were approved by the Institutional Animal Care and Use Committee of Khon Kaen University, based on the Ethic of Animal Experimentation of National Research Council of Thailand (approval number: IACUC-KKU-4/66). Before testing, the mice were kept in an environment with an ambient temperature of 25 °C, and a humidity level of 50–55%, under a cycle of 12 h of light and 12 h of darkness. Animals had unrestricted access to food and water.

The effect of MFE extract on scopolamine-induced memory deficits in mice was determined using Y-maze and water maze tests which were behavioral models. The mice were fed with MFE extract (50, 250, and 500 mg/kg/day) or reference standard donepezil (3 mg/kg/day) by oral administration for 7 days. The mice were investigated for behavioral changes in learning and memory. For both experiments, the animals received the test compounds for 1 h before scopolamine injection. After oral administration, the mice were induced with scopolamine (1 mg/kg) via intraperitoneal (i.p.) for behavioral changes in learning and memory. After thirty minutes, the animals were determined for memory deficit using Y-maze and water maze tests.

The Y-maze test, the Y-maze apparatus, was made of black polypropylene with three equally spaced arms at a 120 angle from each other. Each arm was 40 cm in length, 12 cm in height, 10 and 3 cm in width at the top and bottom, respectively. All mice were placed in the Y-maze apparatus and allowed to run freely through the apparatus for 8 min. After each passage, alcohol (70% ethanol) was used to disinfect the apparatus to completely get rid of the smelly residue the previous mouse left behind. The percentage of alternation was calculated by the manually recorded total number of arm entries and the sequence of entries. The consecutive visits into all three arms without repeated entries were identified as the alternation behavior. The alternation was represented as the ratio of alternation to possible alternation multiplied by 100 [24].

The Morris Water Maze test was used to measure spatial memory. The water maze used a black round pool (70 cm in diameter and 28 cm high). The pool was filled with water (25 °C) and comprised four quadrants (Q1–Q4). At the middle of one quadrant (Q1), the removable platform was immersed 1 cm under the water surface. In the training phase, the mice were trained to memorize the location of platform. The mice were placed in quadrants (Q1–Q4) of the pool and given 1 min to swim around in find of the platform. The time that the mice spent to reach the top of platform was recorded as the escape latency. All animals were performed four tests every day for five straight days. In the test phase, each animal was released to swim in the pool without the platform for 1 min from each quadrant. The time that the animal spent in the area that previously contained the platform (Q1) was recorded as the retention time [24]. 

### 2.8. HPLC Analysis and Validation of the Analytical Method

The HPLC analysis of the phenolic acid and flavonoids were performed with Dionex Ultimate 3000 liquid chromatograph (Germany) using a C18 column (4.6 mm 250 mm) packed with 5 mm diameter particles under gradient conditions. The flow rate of the mobile phase was 0.7 mL/min and consisted of a mixture of solvents: A (acetonitrile) and B (1% aq. acetic acid). The gradient elution was changed from 40% to 60% A in 39 min, and from 60% to 90% B in 50 min. The composition of the mobile phase was restored to its initial condition (solvent A: solvent B: 10: 90) in 55 min. The wavelengths used were 272 nm for gallic acid, ferulic acid, p-coumaric acid, catechin, rutin, and quercetin. Before use, the mobile phase and sample were degassed with an ultrasonic bath after being filtered through a membrane filter at 0.45 m. A concentration range of 5–200 µg/mL of standard stock solutions was prepared in the HPLC mobile phase. The retention time was compared to standards to validate the chromatography peaks [25].

### 2.9. Statistical Analyses

The data were represented as means ± SD (*n* = 5) for in vitro and means ± SEM (*n* = 6) for in vivo experiments. Statistical significance was evaluated by student *t*-test or one-way analysis of variance (ANOVA). For all statistical analyses, a *p*-value < 0.05 was considered significant.

## 3. Results

### 3.1. In Vitro Assay for Activities Related to AD Pathogenesis

The effects of MFE extract on the pathogenic cascade of AD were examined by an in vitro model in the search for a potential candidate for treatment of AD. The total phenolic content and the total flavonoids content of the MFE extract were 109.59 ± 2.12 gallic acid equivalent/g and 43.77 ± 5.92 quercetin equivalent/g. The ABTS and DPPH assays exhibited that the MFE extract had an ability to scavenge ABTS and DPPH radicals with IC_50_ values of 85.28 ± 3.22 and 35.33 ± 0.65 µg/mL, respectively. The acetylcholinesterase inhibitory action was evaluated by using Ellman’s method. The results showed that the MFE extract possessed acetylcholinesterase inhibitory activity with IC_50_ value of 242.49 ± 3.53 µg/mL. Regarding the study of the effect on Aβ aggregation, it was indicated that the MFE extract inhibited Aβ aggregation with IC_50_ value of 39.73 ± 1.72 µg/mL. The results were presented in Table 1.

### 3.2. Neuroprotection against Oxidative Stress

**Figure 1:**
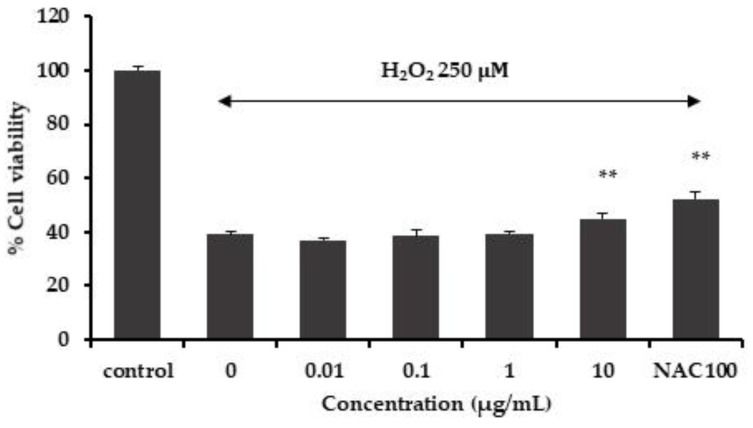
Effect of the MFE extract on hydrogen hydroxide-induced cell death in SH-SY5Y cells. Data are means ± SD (*n* = 5) and ** *p* < 0.01 compared to the H_2_O_2_-treated group.

The MFE extract was evaluated for cytotoxicity on SHSY-5Y cells. The cells were treated with the MFE extract at concentrations of 0.1, 1, 10, and 100 μg/mL for 2 h. The results showed that the MFE extract at the concentration of 0.1–10 μg/mL was non-toxic to SHSY-5Y cells. In order to evaluate the neuroprotective effects in SH-SY5Y cells against cell damage induced by H_2_O_2_, the cells were incubated with MFE extract at concentrations of 0.01, 0.1, 1, and 10 μg/mL. After incubation for 2 h, the cells were incubated with 250 μM H_2_O_2_ for 2 h. The results exhibited that the MFE extract at a concentration of 10 μg/mL significantly inhibited H_2_O_2_-induced neuronal cell death when compared with only the H_2_O_2_-treated group (Figure 1).

### 3.3. Neuroprotection against Aβ_1–42_ Toxicity

**Figure 2:**
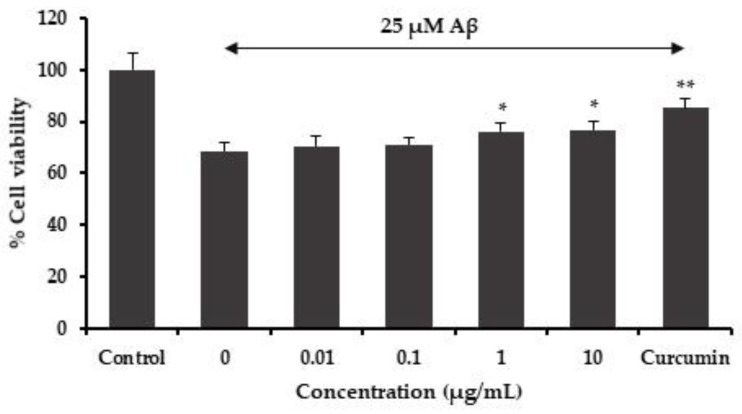
Effect of the MFE extract on Aβ-induced cell damage in SH-SY5Y cells. Data are means ± SD (*n* = 5) and * *p* < 0.05, ** *p* < 0.01 compared to the Aβ-treated group.

The neuroprotective ability of the MFE extract was investigated by using Aβ_1–42_ peptide-induced toxicity in SH-SY5Y cells. The MFE extract showed a neuroprotective effect against Aβ_1–42_ peptide-induced neurotoxicity. The SH-SY5Y cells were pretreated with the MFE extract before Aβ_1–42_ exposure. The results showed that the MFE extracts at concentrations of 1 and 10 μg/mL significantly decreased the cell viability loss generated by the Aβ_1–42_ peptide (Figure 2).

### 3.4. The Effect on the Expression of AD- and Apoptosis-Related Proteins

**Figure 3:**
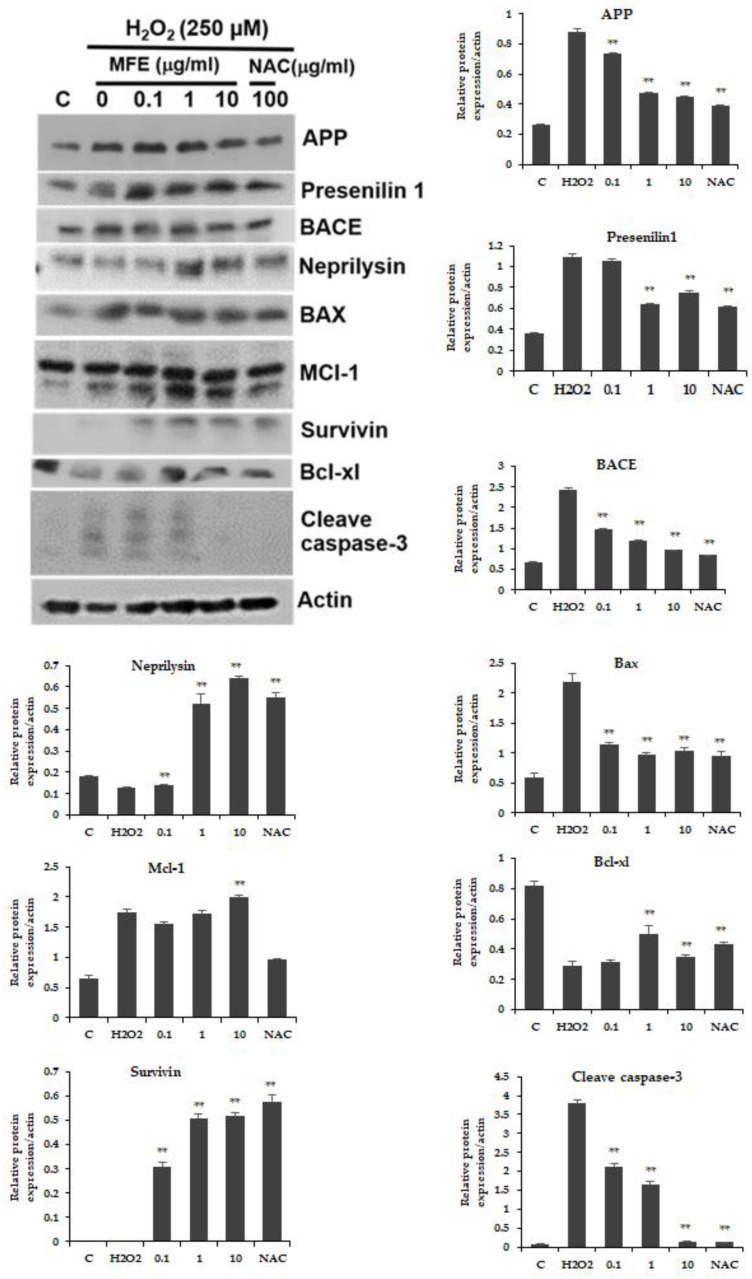
Protein levels of AD-related protein in SH-SY5Y neuroblastoma cells induced with 250 μM H_2_O_2_, with or without pretreatment with MFE extract at 0.1, 1, and 10 µg/mL and N-acetylcysteine (NAC) as standard. Data are means ± SD (*n* = 3) and ** *p* < 0.01 compared to the H_2_O_2_-treated group.

The investigation of the protective mechanisms of the MFE extract against H_2_O_2_-induced cell damage in the SH-SY5Y cell was performed by using Western blot analysis. The cells treated with H_2_O_2_ alone could increase protein levels of amyloid precursor protein (APP), presenilin 1, beta-site APP cleaving enzyme (BACE), Bax, MCl-1, cleaved caspase-3, and decreased expression protein of neprilysin, BCl-xl, and survivin. On the other hand, pretreatment with MFE extract prior to H_2_O_2_ downregulated expression of the pro-apoptotic proteins including cleaved caspase-3 and Bax, while increasing protein levels of MCl-1, BCl-xl, and survivin. In addition, the cells were treated with the MFE extract before the H_2_O_2_ insult, which resulted in an increased protein level of neprilysin and decreased expression of APP, BACE, and presenilin 1. Therefore, the MFE extract showed an ability to protect neuronal damage induced by oxidative stress via the modulating functions of the proteins involved in apoptosis and amyloidogenic pathways (Figure 3).

### 3.5. The Effects of the MFE Extract on Scopolamine-Induced Memory Impairments in Mice

**Figure 4:**
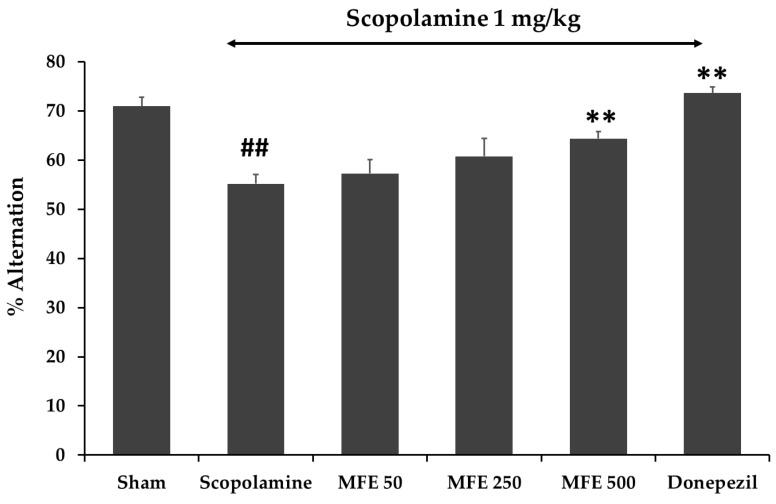
Effect of the MFE extract (50, 250, and 500 mg/kg/day) on memory impairment induced by scopolamine in Y-maze test. Reference standard donepezil (3 mg/kg/day). The data were shown as mean ± SEM (*n* = 6). ## *p* < 0.01 compared with control group (Sham), ** *p* < 0.01 compared with scopolamine-treated group.

A Y-maze test was performed to test short-term spatial working memory. After seven days of MFE treatment, the percentage of alternation behavior was assessed. The result exhibited that the mice in the scopolamine-treated group decrease alternation percentage when compared with the vehicle-treated control group (*p* < 0.01), and the decreased alternation induced by scopolamine (1 mg/kg) was significantly reversed by the MFE extract at dose 500 mg/kg/day (*p* < 0.01) (Figure 4). The result indicated that the flower extract of *M. ferrea* L. enhanced working memory in mice with scopolamine-induced impairment.

**Figure 5:**
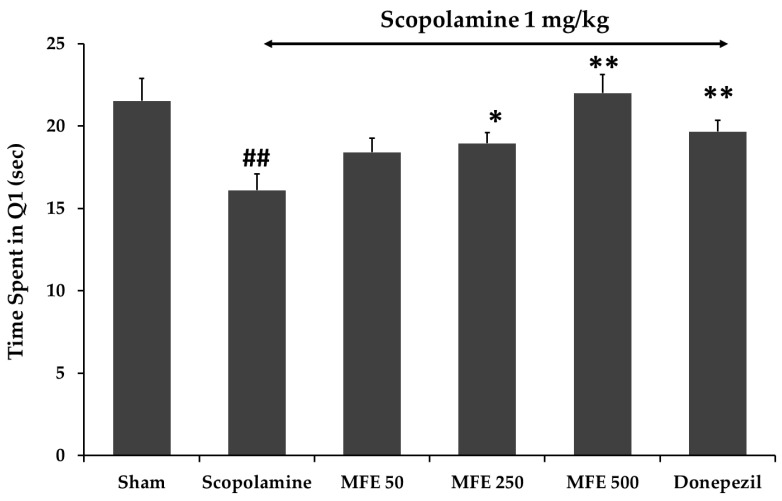
Effect of the MFE extract (50, 250, and 500 mg/kg/day) on memory impairment induced by scopolamine in Morris Water Maze. Reference standard donepezil (3 mg/kg/day). The data were shown as mean ± SEM (*n* = 6). ## *p* < 0.01 compare with control group (Sham), * *p* < 0.05, ** *p* < 0.01 compare with scopolamine-treated group.

The Morris Water Maze (MWM) task was carried out to evaluate the hippocampus-dependent spatial-learning ability. MWM was performed to test long-term memory. Determination of the effect of the MFE extract on scopolamine-induced memory impairment was performed after the mice had been received with MFE extract for seven days. The result exhibited that the mice in the control group spent significantly longer time in quadrant 1 (Q1) than mice in the scopolamine-treated group. The mice that were pretreated with reference standard donepezil (3 mg/kg) significantly improved cognitive deficits indicated by the mice spending longer time in the quadrant (Q1) than the scopolamine-treated mice. In addition, the mice injected with the MFE extract at a dose of 250 and 500 mg/mL also spent significantly more time in the target quadrant than the scopolamine-treated group (Figure 5). The result exhibited that the MFE extract could improve long-term memory in mice with scopolamine-induced amnesia.

### 3.6. HPLC Analysis of the Constituents of the MFE Extract and the Validation Method

**Figure 6:**
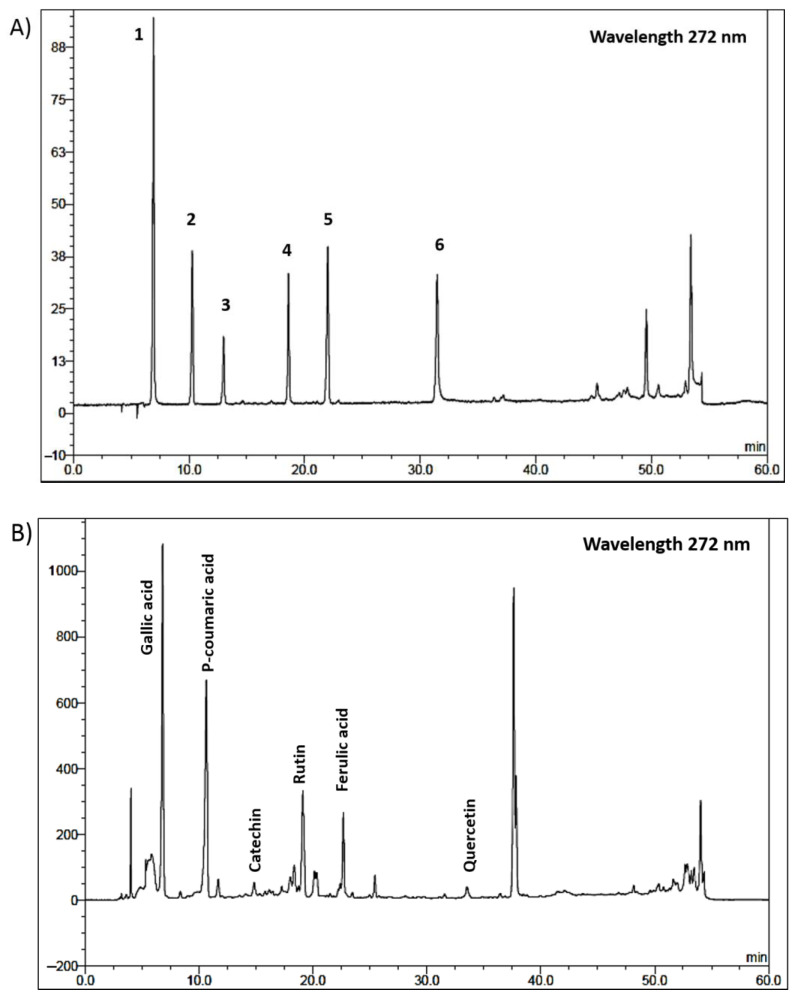
HPLC chromatograms of six standards solution (**A**) and the MFE extract. (**B**) (1 = gallic acid, 2 = p-coumaric acid, 3 = catechin, 4 = rutin, 5 = ferulic acid, 6 = quercetin).

Gallic acid, p-coumaric acid, catechin, rutin, ferulic acid, and quercetin were utilized as markers in the HPLC analysis of the MFE extract. Six different concentrations of standard solutions, i.e., 5–30 µg/mL for gallic acid, 10–60 µg/mL for p-coumaric acid, rutin, ferulic acid, and quercetin, 20–100 µg/mL for catechin, were used. The range, linearity, LOD, LOQ, precision, and accuracy of the HPLC technique were determined as validation parameters. The validation results revealed good linearity with the coefficient of determination (r^2^) greater than 0.99, high precision with low percentage relative standard deviation and good accuracy with percentage recovery rates within 90–110%. Thus, the developed HPLC method is appropriate for the analysis of the six chemicals in the MFE extract (Table 2). The HPLC method was used to determine the amount of gallic acid, coumaric acid, catechin, rutin, ferulic acid, quercetin in the crude extract, and the retention time shown in Figure 6 was consistent with the standard solutions and the MFE extract. The amount of gallic acid, coumaric acid, catechin, rutin, ferulic acid, and quercetin in the MFE extract were 13.17 µg/mg extract, 17.18 µg/mg extract, 2.73 µg/mg extract, 3.06 µg/mg extract, 1.91 µg/mg extract, and 0.34 µg/mL, respectively.

## 4. Discussion

Several phytochemical studies have shown that the chemical components of *M. ferrea* L. flower are phenolic acid [13], flavonoid [13], coumarin [14] and xanthone [13,15]. A previous study reported the anti-AD action of phenolic acids which prevented the development of AD pathology by inhibiting the Aβ aggregation pathway [26]. Flavonoids have been revealed to possess multiple biological activities related to AD pathology including anti-acetylcholinesterase, anti-Aβ aggregation, antioxidant, and anti-inflammation [27]. In this study, the flavonoids (rutin, catechin, quercetin) and phenolic acids (gallic acid, ferulic acid, p-coumaric acid) in MFE extract were quantified by using HPLC. Several studies have shown that these compounds exhibited antioxidant [28,29,30], anti-AChE function [31,32,33], anti-Aβ aggregation [34,35,36,37,38], and neuroprotection [39,40,41,42]. Our HPLC results showed that the major phenolic acids found in the MFE extract were p-coumaric acid and gallic acid, while the major flavonoid was rutin. Previous studies have showed that p-coumaric acid and gallic acid possessed neuroprotective action against Aβ toxicity [38,40].

The cholinergic system is well known to be important for a variety of cognitive functions [43]. According to evidence, cognitive impairment in AD is associated with a deterioration of acetylcholine (ACh) transmission, which plays important roles in learning and memory functions [44]. Cholinergic transmission is terminated when ACh is hydrolyzed by AChE. Thus, AChE inhibition is a crucial target for AD therapy. This study has established that the MFE extract had an ability to inhibit AChE function in a dose-dependent manner. A prior investigation demonstrated that various phenolic acids and flavonoids possessed an AChE inhibitory action [45]. In addition, gallic acid and p-coumaric acid, which are the major components in the MFE extract, showed an ability to inhibit AChE activity [31]. Thus, anti-AChE activity of the MFE might partly come from gallic acid, p-coumaric acid, and rutin, which are the major components found in the extract.

Numerous studies indicated that oxidative damage was found in the brains of patients with AD and may contribute to the onset of AD [46]. The imbalance between the production and the removal of free radicals is reflected in oxidative stress. [47]. Therefore, antioxidant drugs can interrupt the progression of AD by preventing the neurotoxic and damaging consequences of oxidative stress. In this investigation, the MFE extract exhibited antioxidant activities in in vitro models. A prior research approach indicated high correlations between total phenolic, flavonoid content, and radical scavenging activity [48]. Heim and coworkers reported that rutin, catechin, and quercetin showed an ability to scavenge free radicals by an ABTS assay [30]. Several studies revealed that p-coumaric acid, gallic acid, and ferulic acid exhibited antioxidant properties determined by DPPH, ABTS, FRAP, and ORAC [49,50]. From the HPLC analysis, the MFE extract contains various flavonoids (rutin, catechin, quercetin) and phenolic acids (gallic acid, ferulic acid, p-coumaric acid), thus MFE showed an ability to scavenge free radicals via the action of these flavonoids and phenolic components.

The deposit of Aβ plaques in the brains of AD patients is a hallmark of AD pathogenesis. Hence, preventing or lowering the aggregation of Aβ has become the main approach of many therapeutic strategies that have been developed or presently are in clinical trials. A major component of amyloid plaques in AD brains is Aβ_1–42_. Several studies have shown that Aβ_1–42_ plays a role in the etiology of AD [51,52]. In addition, the generation of Aβ_1–42_ was found to increase in familial AD patients [53]. Studies using transgenic mice and in vitro models showed that Aβ_1–42_ might form amyloid plaques faster than Aβ_1-40_. So, to evaluate the Aβ aggregation and the Aβ-induced cytotoxicity in SH-SY5Y neuronal cells, we used Aβ_1–42_. In this study, we used the ThT fluorescence assay to evaluate the effects of MFE extracts on the inhibition of Aβ aggregation. The results exhibited that the MFE extract could inhibit the aggregation of Aβ_1–42_. Additionally, it has been shown that Aβ is harmful to neurons via a number of mechanisms, including mitochondrial malfunction, ROS production, and apoptosis [54,55]. Thus, the neuroprotective effect of the MFE extract against Aβ_1–42_ toxicity in SH-SY5Y cells was also investigated in the present study. Our results showed that Aβ_1–42_ could induce neuronal cell death. This result was consistent with a previous study, indicating that Aβ_1–42_ at micromolar concentration levels caused neuronal death in culture cells [56]. Pretreatment of the SH-SY5Y cells with the MFE extracts at concentrations of 1 and 10 μg/mL significantly decreased the cell viability loss generated by the Aβ_1–42_ peptide. Thus, the MFE extract showed a neuroprotective effect against Aβ_1–42_ peptide-induced neurotoxicity.

Reactive oxygen species (ROS) can destroy neuronal cells via apoptosis. Exogenous ROS sources such as H_2_O_2_ are frequently employed to induce apoptosis [57]. A previous report showed etiological associations between neurodegenerative diseases and the production of H_2_O_2_ [58]. As a result, H_2_O_2_-induced neuronal damage is thought to be a viable model for studying neurodegeneration brought on by oxidative stress [59]. In this study, the vitality of cells exposed to H_2_O_2_ was significantly increased by pretreating SH-SY5Y cells with the MFE extract. For neuroprotective action, the antioxidant properties of the MFE extract may contribute to its neuroprotective effects. In addition to the antioxidant effect, the protective effect can result from other mechanisms. Thus, to determine the protective mechanisms of the MFE extract against H_2_O_2_-induced cell death, we used Western blot analysis to identify the MFE extract that affected the expression of pro-apoptotic and anti-apoptotic proteins. Pretreatment of SH-SY5Y cells with the MFE extract before H_2_O_2_ exposure showed downregulation of the pro-apoptotic proteins including Bax and cleaved caspase-3 and upregulation of the anti-apoptotic proteins including BCl_xl_, MCl_1_, and survivin. Previous research studies revealed that the mitochondrial apoptotic pathway was associated with oxidative stress-induced neuronal cell death in H_2_O_2_-induced SH-SY5Y cells, which was a key mechanism in neurodegenerative diseases [60]. The permeability of the mitochondrial membrane was increased as a result of excessive ROS production, causing cytochrome C to be released. Cytochrome C release stimulates pro-apoptotic factors, such as caspase-3 and causes eventual cell death. Our results indicated that, after being treated with H_2_O_2_, proteolytic cleavage of caspase-3 increased and pro-apoptotic proteins of Bax also increased. The MFE extract protected neuronal cells against H_2_O_2_-induced death by downregulation of cleaved caspase-3, decreased pro-apoptotic protein of Bax, as well as increased expression of MCl_1_, BCl_xl_, and survivin. Therefore, the antioxidant and anti-apoptotic properties of the MFE extract appear to prevent neuronal cell death brought on by oxidative stress.

Furthermore, the accumulation of Aβ plaques in AD brains was believed to be partly enhanced by oxidative stress. Several studies had demonstrated the relationship between oxidative stress and Aβ plaques development. Oxidative stress played important roles in the progression of Aβ peptides via the regulation of APP metabolism [61]. According to the amyloid cascade theory, the neurotoxic amyloid peptides that were the hallmark of AD could be produced as a result of the proteolytic cleavage of APP [51]. There are two APP processing pathways, including amyloidogenic or nonamyloidogenic pathways. In the nonamyloidogenic pathway, APP is sequentially cleaved by α- and γ-secretases to generate a soluble small peptide that does not have neurotoxic effects. In the harmful amyloidogenic pathway, the sequential cleavage of APP by β- and γ-secretase promote a long chain insoluble Aβ. The Aβ will aggregate to form an amyloid plaque and accumulate outside the cell resulting in cell death.

In the present study, the effect of the MFE extract on the expression of AD-related proteins was investigated by Western blotting analysis. The expression levels of proteins involving Aβ production (presenilin 1, BACE, and APP) and Aβ clearance (neprilysin) were studied. The neuronal cells induced with only H_2_O_2_ were found to result in the upregulation of presenilin 1, BACE, and APP compared to untreated normal cells. However, pretreatment with the MFE extract prior to H_2_O_2_ alleviated the expression of presenilin 1, BACE, and APP. Presenilin 1 is a subunit of the γ-secretase. It was considered to play an important role in the production of Aβ from APP to provide Aβ fragments and that its absence or inhibition could decrease the production of Aβ [62]. In AD research, this has been proposed as a potential target for an anti-amyloidogenic strategy [63]. Furthermore, the β-secretase that is involved in the processing of APP to create Aβ includes BACE1 and its homolog, BACE2 [64]. It has been demonstrated that BACE1 is a more powerful inducer of APP cleavage leading to the production of Aβ [65]. In an in vitro model, BACE2 was effective at cleaving APP at the secretase cleavage site [66] and releasing Aβ in mutant transfected cells [67]. Moreover, in a model of transgenic mice with an APP mutation, BACE overexpression accelerated APP processing and resulted in elevated levels of Aβ [68]. This is in line with research showing that AD patients’ brains have increased BACE activity due to the presence of a high Aβ plaque load [69,70]. For the Aβ clearance protein, the expression of neprilysin tended to decrease after H_2_O_2_ treatment when compared with the control, whereas the cells treated with the MFE extract prior to the H_2_O_2_ led to an increase in neprilysin protein expression. According to the report, neprilysin promoted the hydrolysis of Aβ [71]. Neprilysin protein and mRNA levels were found to be considerably reduced in the hippocampus region of AD patients compared to controls [72]. Therefore, the decrease in neprilysin levels may affect the Aβ clearance ability of neuronal cells, leading to the deposition of Aβ in AD brains [73]. Accordingly, neprilysin protein regulation with the MFE extract revealed that the MFE extract might prevent Aβ accumulation which is partly mediated by clearance of Aβ. Conclusively, our results indicated that the MFE extract could protect neurons by preventing Aβ accumulation via inhibition of Aβ production and acceleration of Aβ clearance.

Subacute toxicity of the ethanolic extract of *Mesua ferrea* L. flowers in rats showed that at the high dose, it did not have any serious toxicity [74]. In addition, we studied the effect of the MFE extract on memory deficit induced by scopolamine in ICR mice. Scopolamine, a nonselective muscarinic antagonist, is widely used to reduce memory and learning abilities. Scopolamine-induced amnesia is the classical method to study the effects of novel cognition-enhancing drugs in animals [75]. The Y-maze test was used to evaluate short-term memory or working memory by examining the animal’s spontaneous alternation behavior. The mice were eager to explore the new arm of the maze as their normal behavior, rather than going back to the one they have already explored. In contrast, the mice with impaired short-term memory failed to recognize which arm they had already been in that was presented by a decreased spontaneous alternation. The Morris Water Maze was used to test long-term memory. It was widely used to study spatial memory and learning abilities [76]. The mice that spent more time during the testing day in the target quadrant where the platform had been located during the training days, represent an improvement in spatial memory. From this study, memory loss in amnesic mice was significantly induced by scopolamine. The seven-day MFE extract treatment reversed the memory deficit induced by scopolamine. The mice treated with the test compound showed a significantly increased percentage of alternation in the Y-maze test and time spent in the target quadrant (Q1) in the water maze test, illustrating an enhancement in short-term and long-term memories, respectively. According to several studies, scopolamine decreased the effectiveness of the cholinergic system by acting on muscarinic receptors and increasing AChE activity [77]. From an in vitro AChE activity determination, the MFE extract showed AChE inhibitory activity. The MFE extract reversed the effect of scopolamine-induced memory loss, indicating that their mechanisms of action were on the same target. Therefore, the cognitive improvement resulting from the MFE extract treatment was contributed by the AChE inhibitory action.

## 5. Conclusions

The results of the present study demonstrated that the MFE extract had a variety of mechanisms of action that were directed at various targets in the AD pathogenesis cascade which included free radical scavenging, AChE and Aβ aggregation inhibitory actions, as well as neuroprotection against H_2_O_2_ and Aβ toxicity. The MFE extract exhibited neuroprotection via the downregulation of the pro-apoptotic protein, specifically, the following: blocking Bax and caspase-3 cleavage activation while upregulating the following anti-apoptotic proteins: MCl_1_, BCl_xl_, and survivin. Moreover, the MFE extract might have an ability to inhibit the expression of proteins involving Aβ production (APP, presenilin 1, and BACE) and might accelerate the Aβ clearance protein (neprilysin). Overall, results showed that the MFE extract has several modes of action related to the AD pathogenesis cascade including antioxidants, anti-acetylcholinesterase, anti-Aβ aggregation, and neuroprotection against oxidative stress and Aβ-. In addition, the MFE extract showed an ability to enhance scopolamine-induced memory impairment in mice. Therefore, the *M. ferrea* L. flower extract might have a potential for further development as a medication for AD.

## Figures and Tables

**Table 1:** The chemical content and the activities related to AD of the MFE extract.

Index	MFE Extract (IC_50_) μg/mL	Trolox (IC_50_) μM	Tacrine (IC_50_) μM	Qurcumin (IC_50_) μM
Total phenolic content, mgGAE/g Extract	109.59 ± 2.12	ND	ND	ND
Total flavonoid content, mgQE/g Extract	43.77 ± 5.92	ND	ND	ND
Antioxidant ABTS assay	85.28 ± 3.22	64.83 ± 0.77	ND	ND
Antioxidant DPPH assay	35.33 ± 0.65	25.00 ± 0.57	ND	ND
AChE inhibitory	242.49 ± 3.53	ND	0.40 ± 1.3	ND
Anti-Aβ aggregation	39.73 ± 1.72	ND	ND	3.01 ± 2.35

ND = Not detected.

**Table 2:** Validation results of the analytical method for determination of gallic acid (1), p-coumaric acid (2), catechin (3), rutin (4), ferulic acid (5), quercetin (6) content in the MFE extract.

Parameter				Compounds			
		Gallic Acid	Coumaric Acid	Catechin	Rutin	Ferulic Acid	Quercetin
**Linearity**	Range (µg/mL)	5–30	10–60	20–100	10–60	10–60	10–60
	Coefficient Determination (R^2^)	0.9982 ± 0.00032	0.9960 ± 0.00049	0.9978 ± 0.00036	0.9959 ± 0.00006	0.9909 ± 0.00070	0.9926 ± 0.00107
**LOD (µg/mL)**		0.1	0.1	0.2	0.3	0.2	0.5
**LOQ (µg/mL)**		0.2	0.5	0.5	0.9	0.7	1.3
**Precision (%RSD)**	Within day	0.19–0.83	0.69–1.99	0.54–1.50	0.22–0.89	0.25–1.46	0.67–1.18
	Between day	0.16–1.10	0.39–1.32	0.47–1.96	0.43–2.74	0.04–0.49	0.90–3.14
**Accuracy (%)**	Conc. (Low)	102.58	101.65	107.19	109.73	107.18	101.37

## Data Availability

All the data generated or analyzed during this study were included in this published article.
